# The role of embodiment in the treatment of patients with anorexia and bulimia nervosa: a 2-year follow-up study proposing an integration between enhanced cognitive behavioural therapy and a phenomenological model of eating disorders

**DOI:** 10.1007/s40519-021-01118-3

**Published:** 2021-02-03

**Authors:** Eleonora Rossi, Giovanni Castellini, Emanuele Cassioli, Carolina Sensi, Milena Mancini, Giovanni Stanghellini, Valdo Ricca

**Affiliations:** 1 Psychiatry Unit, Department of Health Sciences, University of Florence, Florence, Italy; 2 Department of Psychological, Health and Territorial Sciences, G. D’Annunzio University, Chieti, Italy

**Keywords:** Anorexia nervosa, Bulimia nervosa, Embodiment disorder, Enhanced cognitive behavioural therapy, Phenomenology

## Abstract

**Purpose:**

Recent studies demonstrated that the embodiment disorder represents a core feature of eating disorders (EDs). The aim of this study was to evaluate the role of its variation as a possible mediator of the efficacy of enhanced cognitive behavioural therapy (CBT-E) on classic ED symptomatology, including body uneasiness.

**Methods:**

73 patients with anorexia nervosa and 68 with bulimia nervosa were treated with a multidisciplinary approach including CBT-E. Psychometric questionnaires were administered at baseline (T0) and after one (T1) and 2 years (T2) to evaluate general and ED-specific psychopathology, body uneasiness and the embodiment disorder. Data regarding diagnostic crossover and remission were also collected.

**Results:**

Longitudinal analysis showed an improvement of all psychopathological dimensions at T1, which remained stable at T2 (*p* < 0.05). Remission rate at T2 was 44.7%, and diagnostic crossover occurred in 17.0% of patients. Higher levels of embodiment disorder predicted increased diagnostic instability (OR: 1.80 [1.01–3.20], *p* = 0.045). The amelioration of the embodiment disorder mediated the decrease in both ED-specific psychopathology (indirect effect: 0.67 [0.46–0.92]) and body uneasiness (indirect effect: 0.43 [0.28–0.59]).

**Conclusion:**

For the first time, these findings highlighted the role of the embodiment disorder as a maintaining factor of ED symptomatology, supporting the importance of integrating CBT-E with a phenomenological model of EDs.

**Level of evidence:**

Level IV, longitudinal observational study (case series).

## Introduction

Anorexia nervosa (AN) and bulimia nervosa (BN) are complex eating disorders (EDs) that mainly affect young women [1].

According to the transdiagnostic cognitive-behavioural model of EDs, these disorders share a common psychopathological core represented by the excessive importance attributed to body shape and weight [2]. Indeed, patients with EDs structure their self-esteem primarily, or even exclusively, based on their shape and weight and their ability to control them.

Around this core psychopathological feature has converged the attention of both cognitive, psychodynamic and phenomenological scholars [3]. Indeed, a new phenomenological perspective has emerged, according to which the classic symptoms of EDs would be epiphenomena of a more profound core phenomenon represented by a disorder of the embodiment [4, 5]. The concept of “embodiment” refers to how people experience their own body, overcoming the simple involvement of the visual perception of the body by including coenaesthesia, that is the direct, pre-reflective apprehension of one’s corporeality in the first-person perspective.

According to the optical-coenaesthesic disproportion hypothesis of EDs [6–8], the embodiment disorder could be operationally defined as the result of two components: a deep affection of coenaesthesia and the consequent loss of the capacity of experiencing one’s own body “from within” and, as a form of compensation to it, an over-reliance on the gaze of the others as a way through which feeling oneself as an embodied self. The body, exposed and subjected to the other’s gaze, is thus reduced to its appearance, and it becomes just an *object seen by the others* [9]. Most of the typical symptoms of EDs might be the consequence of this affection of lived corporeality, such as the overvaluation of the importance of the objective bodily dimensions (i.e., weight) and of the capacity of starving and exerting control over dietary intake. Over time, patients tend to identify themselves with these behaviours, which become devices through which they can define themselves and shape their identity.

One of the psychopathological features of EDs that seems to be more resistant to treatment is body uneasiness [10, 11]. This feature consists in a cognitive-affective attitude toward one’s own body that embraces various concepts related to negative body image, such as body dissatisfaction, avoidance or, on the contrary, compulsive control of one’s own body, detachment and estrangement feelings towards body and worries about particular body parts, shapes or functions [12]. Several studies showed that various components of body image dissatisfaction and uneasiness in patients with EDs, other than being a frequent residual symptom after treatment, as previously stated, are associated with a worse prognosis [2, 13, 14] and with a higher likelihood of relapse after remission [15, 16]. Therefore, body uneasiness represents a key focus in the treatment of patients with AN and BN.

At present, the guidelines of the National Institute for Health and Care Excellence (NICE) recommend Cognitive Behavioural Therapy-Enhanced (CBT-E) as a first-line treatment in managing AN and BN in adults [17]. Indeed, many studies demonstrated the efficacy of CBT-E in the treatment of these disturbs [18–20]. However, available data show that the outcome of patients with AN or BN treated with CBT-E is far from being satisfactory, with a long- term remission rate which is less than 50% [21, 22].

These unsatisfactory data regarding the long-term outcome of EDs might be the consequence of the paucity of evidence regarding the mediators of the efficacy of treatment interventions on EDs psychopathology, both in terms of body uneasiness and of overvaluation of body shape/weight [23, 24].

In light of these considerations, the present study was designed as a naturalistic and longitudinal observation of patients with AN and BN treated with a multidisciplinary therapy which included CBT-E. Moving from the phenomenological perspective on EDs psychopathology, the primary hypothesis was that a disordered embodiment represents a *core feature* and a *maintaining factor* of EDs symptoms. In particular, considering the increasingly acknowledged role of the embodiment disorder as a deeper layer in the conceptualization of EDs [4, 5], it could be hypothesised that an improvement of this psychopathological feature might mediate the reduction of classic ED symptoms. Thus, the aim of the present study was to evaluate the role of the variation of the embodiment disorder as a possible mediator of the efficacy of CBT-E on the overvaluation of body shape/weight and body uneasiness.

## Methods

The present observational longitudinal study with a 2-year follow-up was performed at the Outpatient Clinic for Eating Disorders of the University of Florence. During the first evaluation, the procedures of the study were explained. After that, patients were asked to provide their written informed consent. The Ethics committee of the institution approved the study protocol.

### Participants

Patients were enrolled in the study between February 2016 and April 2018, provided they met the following inclusion criteria: female gender, age between 18 and 40 years and current DSM-5 diagnosis of AN or BN. Exclusion criteria were as follows: comorbid schizophrenia, bipolar I disorder, illiteracy, intellectual disability, severe medical conditions that precluded an outpatient treatment, such as severe heart, renal, or liver failure, current use of psychoactive medications, except for antidepressants and benzodiazepines, which were kept stable across the longitudinal observation. Of the 192 AN and BN patients referred, 7 subjects declined to participate, and 11 were excluded (1 comorbid schizophrenia, 1 comorbid bipolar disorder, 2 intellectual disability, 3 severe medical conditions, 4 concurrent use of psychoactive medication). Only those patients who performed both baseline and the two follow-up assessments were included in the study.

### Study design

Data collection was performed during the first outpatient visit (baseline, T0), 1 year (T1) and 2 years (T2) after the baseline evaluation. Dropout was defined as failing to continue treatment for any reason.

### Assessment

Two expert psychiatrists performed the initial clinical assessment. Clinical, pharmacological, and sociodemographic data were collected through a face-to-face interview, during the first outpatient visit and at the follow-up assessments. Standard calibrated instruments were used for the anthropometric measurements, and BMI was calculated as weight in kilograms divided by height in squared meters. Psychopathological features were studied using several self-reported questionnaires, including:

- Eating Disorder Examination Questionnaire version 6.0 (EDE‐Q 6.0) [2]: to assess the core psychopathology of EDs. This questionnaire provides four subscales: dietary restraint, eating concern, weight concern and shape concern. A total score (EDE-Q TS) can be obtained by averaging the score reported in each subscale.
- Body Uneasiness Test-A (BUT-A) [12]: a questionnaire that evaluates body uneasiness. It consists of 34 items, providing five subscales: weight phobia, body image concern, avoidance, compulsive self‐monitoring and depersonalization. A total score (Global Severity Index, BUT GSI) can be obtained by averaging the scores reported in all the items.
- Identity and Eating Disorders (IDEA) [4]: a questionnaire evaluating identity and embodiment in patients with EDs. It consists of 23 items divided into four subscales: feeling oneself only through the gaze of the other and defining oneself only through the evaluation of the other (IDEA GEO); feeling oneself only through objective measures (IDEA OM); feeling extraneous from one’s own body (IDEA EB); feeling oneself only through starvation (IDEA S). The average of all items represents the total score (IDEA TS). Higher scores indicate a severer disorder of the embodiment.
- Symptom Checklist 90-Revised (SCL 90‐R) [25]: a questionnaire assessing the general psychopathology that provides a global severity index (GSI) obtained by averaging the scores reported in all the items.
  During the follow-up visits, data were collected regarding the following outcome variables:
- Diagnostic crossover: defined as not meeting full criteria for the ED initially diagnosed at T0, while the criteria for a different ED are met.
- Remission: following the definitions contained in the DSM-5 [1], a patient obtained a full remission when none of the diagnostic criteria for the initially diagnosed ED was met for a sustained period, while a partial remission was obtained when only some of the criteria persist (for AN the persisting criteria cannot include underweight).

### Treatment

All recruited patients received a multidisciplinary treatment that included individual CBT-E for at least 40 weeks, applying validated cognitive and behavioural strategy for the treatment of EDs [2]. Moreover, regular dietetic evaluations were performed, and specialist medical examinations were carried out when necessary. Nine therapists experienced in the treatment of individuals with EDs took part in the present study. Expert psychiatrist and psychotherapists performed weekly supervision meetings to monitor the correct implementation of treatments. Moreover, treatment included regular dietetic and psychiatric evaluations and, when necessary, medical visits. The treatment took place initially in the context of Day Hospital and subsequently of regular outpatient visits.

### Statistical analyses

Comparisons between groups (AN vs. BN) were performed using independent-samples *t*-tests. Longitudinal course of the parameters taken into consideration was analysed using Linear Mixed Model with random intercepts, inserting Time, Diagnosis and Time * Diagnosis as fixed effects; the Time * Diagnosis interaction was included in the model to study the possible moderation effect of the diagnosis on the relationship between Time and the variables under study. Multivariate binomial logistic regression analysis was used to test whether baseline IDEA TS significantly predicted outcome variables (diagnostic crossover, remission) while controlling for the initial ED diagnosis; results were reported as odds ratios (ORs) with a 95% confidence interval.

A two-instance repeated-measures mediation analysis was performed using the MEMORE macro for SPSS [26] to test whether Time affected ED-specific psychopathology and body uneasiness through a variation of the embodiment disorder. Two similar models were run, with Time as a within-subjects independent variable, embodiment (IDEA TS) as a mediator, and ED-specific psychopathology (EDE-Q TS) and body uneasiness (BUT GSI) as dependent variables. Bootstrapping with 20,000 resamples was used to estimate a 95% confidence interval (CI) for indirect effects: an indirect effect is considered statistically significant if the CI excludes zero. Since the estimated relationship between the mediator and the dependent variable could not be assumed to be equal across instances of the independent variable in the two-instance repeated-measures mediation model, the interaction between Time and the mediator were included in the models. This meant including the average of the variation of the mediator in the model, in addition to the difference [26]. Before proceeding with the final model, the necessary conditions were verified for a mediating effect to occur in a repeated-measures design [27].

## Results

### Baseline characteristics of the sample

Of the 174 patients recruited who completed the baseline assessment, 33 were lost to follow-up (16 dropouts, 17 were not able to perform follow-up visits). No significant differences were detected at baseline regarding the psychopathological and clinical features between completers and lost-to-follow-up patients.

The final sample consisted of 141 patients with EDs, 73 with AN (34 of the restricting subtype and 39 of the binge-purging subtype) and 68 with BN. The mean age was 25.73 ± 10.41 and 30.62 ± 11.42 years for participants with AN and BN, respectively (*p* = 0.01). Patients with BN were found to have a longer duration of illness than patients with AN (12.44 ± 10.86 and 6.79 ± 8.97 years, respectively; *p* = 0.005). A total of 24 (17.0%) patients received pharmacological therapy to treat concurrent psychiatric symptomatology: 23 received a selective serotonin reuptake inhibitor (SSRI) (of which 3 received fluoxetine), and 5 received benzodiazepines. BMI and psychometric scales data collected at baseline are reported in Table 1.

**Table 1:** Longitudinal trend of clinical and psychometric measurements

	Anorexia nervosa *n* = 73			Bulimia nervosa *n* = 68			Time effect (*F*)	Diagnosis effect (*F*)	Time*Diagnosis Interaction effect (*F*)
	T0	T1	T2	T0	T1	T2			
BMI (kg/m^2^)	16.25 ± 1.44	17.98 ± 1.90^†^	18.10 ± 2.20^†^	24.28 ± 5.26	24.73 ± 5.84	24.53 ± 5.23	8.08***	124.62***	15.90***
SCL-90-R GSI	1.46 ± 0.74	1.03 ± 0.77^†^	0.90 ± 0.71^†^	1.61 ± 0.75	1.10 ± 0.70^†^	1.14 ± 0.78^†^	37.60***	1.78	0.15
BUT-A									
GSI	2.26 ± 1.17	1.82 ± 1.24^†^	1.69 ± 1.21^†^	2.60 ± 1.10	2.00 ± 1.06^†^	1.95 ± 1.12^†^	27.63***	2.68	0.46
Weight phobia	2.81 ± 1.43	2.35 ± 1.45^†^	2.18 ± 1.54^†^	3.21 ± 1.18	2.73 ± 1.19^†^	2.64 ± 1.31^†^	19.09***	3.98*	0.11
Body image concerns	2.48 ± 1.29	1.93 ± 1.36^†^	1.90 ± 1.36^†^	3.02 ± 1.35	2.32 ± 1.28^†^	2.31 ± 1.28^†^	25.91***	5.27*	0.40
Avoidance	1.50 ± 1.13	1.39 ± 1.25	1.20 ± 1.14	2.10 ± 1.43	1.44 ± 1.11^†^	1.39 ± 1.26^†^	12.24***	2.81	4.21*
Compulsive self-monitoring	2.18 ± 1.36	1.80 ± 1.26^†^	1.63 ± 1.20^†^	1.99 ± 1.35	1.60 ± 1.17^†^	1.46 ± 1.08^†^	13.75***	0.45	0.15
Depersonalization	2.02 ± 1.23	1.41 ± 1.21^†^	1.26 ± 1.17^†^	2.18 ± 1.28	1.47 ± 1.29^†^	1.47 ± 1.24^†^	26.68***	0.89	0.04
EDE-Q									
Total score	3.01 ± 1.72	2.02 ± 1.66^†^	2.04 ± 1.74^†^	3.54 ± 1.37	2.52 ± 1.45^†^	2.53 ± 1.61^†^	35.19***	5.38*	0.01
Restraint	3.25 ± 2.07	1.70 ± 1.69^†^	1.95 ± 1.96^†^	3.04 ± 1.75	2.18 ± 1.65^†^	2.15 ± 1.90^†^	27.76***	0.74	3.30*
Eating concerns	2.53 ± 1.60	1.61 ± 1.50^†^	1.62 ± 1.52^†^	3.13 ± 1.62	1.98 ± 1.57^†^	2.05 ± 1.78^†^	32.11***	4.13*	0.30
Weight concerns	2.95 ± 1.81	2.18 ± 1.87^†^	2.13 ± 1.88^†^	3.81 ± 1.56	2.81 ± 1.66^†^	2.80 ± 1.72^†^	22.07***	8.37**	0.31
Shape concerns	3.32 ± 1.89	2.59 ± 1.94^†^	2.47 ± 1.97^†^	4.19 ± 1.61	3.09 ± 1.73^†^	3.13 ± 1.93^†^	23.32***	7.12**	0.97
IDEA									
Total score	1.73 ± 0.91	1.41 ± 1.02^†^	1.20 ± 0.99^†^	1.75 ± 0.78	1.31 ± 0.86^†^	1.32 ± 0.90^†^	22.57***	0.01	0.10
GEO	1.47 ± 1.09	1.28 ± 1.10	1.06 ± 1.06^†^	1.60 ± 1.04	1.25 ± 0.95^†^	1.22 ± 1.03^†^	10.39***	0.14	0.12
OM	2.15 ± 1.15	1.72 ± 1.25^†^	1.51 ± 1.30^†^	2.29 ± 1.00	1.73 ± 1.01^†^	1.66 ± 1.17^†^	17.71***	0.44	0.06
EB	1.41 ± 1.09	1.07 ± 1.12^†^	0.81 ± 0.80^†^	1.49 ± 0.99	0.94 ± 1.05^†^	1.12 ± 0.99^†^	14.63***	0.25	0.48
S	2.21 ± 1.00	1.71 ± 1.02^†^	1.61 ± 1.23^†^	1.77 ± 1.07	1.39 ± 0.99	1.35 ± 0.96^†^	17.25***	4.05*	1.32

For each score, a statistical analysis was carried out using Linear Mixed Model, inserting Time, Diagnosis and Time*Diagnosis as Fixed Effects, with random intercepts. The table reports means and standard deviations for each timepoint, and the test results relating to the Fixed Effects (F)

*AN* anorexia nervosa; *BN *bulimia nervosa; *BUT-A* Body Uneasiness Test-A; *EB* feeling extraneous from one’s own body; *EDE-Q* Eating Disorder Examination Questionnaire; *GEO* feeling oneself only through the gaze of the other and defining oneself only through the evaluation of the other; *GSI *Global Severity Index; *IDEA* Identity and Eating Disorders; *OM *feeling oneself only through objective measures; *S *feeling oneself only through starvation; *SE *standard error; *SCL-90-R* Symptom Checklist 90-Revised

*p < 0.05; **p < 0.01; ***p < 0.001; †*p* < 0.05 for the Bonferroni-corrected post-hoc pairwise comparison with T0

Subjects affected by BN had higher scores in the body image concerns (*p* = 0.017) and avoidance (*p* = 0.006) subscales of the BUT questionnaire and in almost all EDE-Q scores (all *p* < 0.050, except for EDE-Q Dietary Restraint) as compared to those with AN, and lower scores in the Starvation subscale of the IDEA questionnaire (*p* = 0.012).

### Longitudinal analysis

**Fig. 1:**
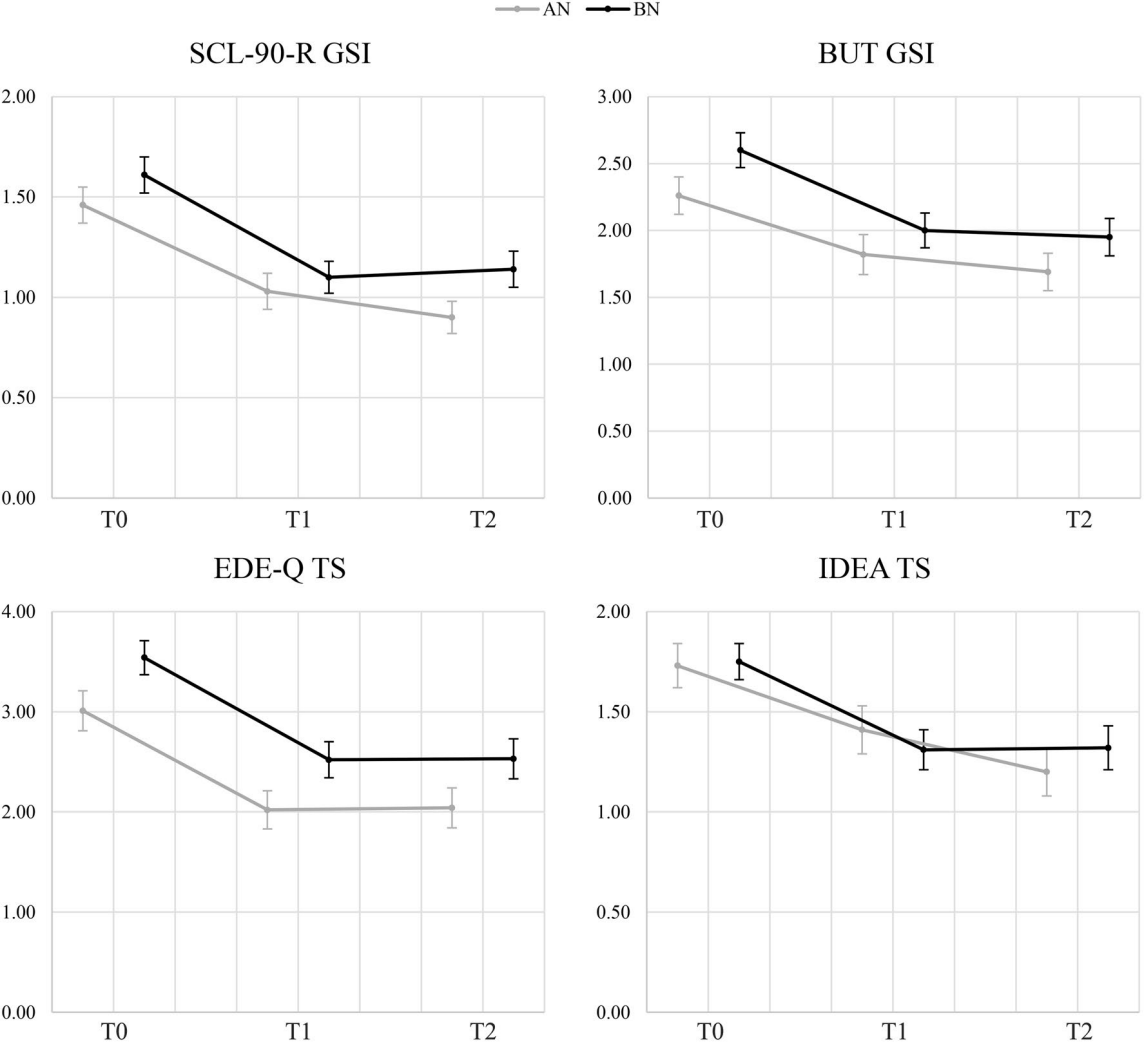
Longitudinal course of general (SCL-90-R GSI) and ED specific (EDE-Q TS) psychopathology, body uneasiness (BUT GSI) and embodiment disorder (IDEA TS), divided by diagnosis. Standard errors are reported as error bars. AN anorexia nervosa; *BN* bulimia nervosa; *BUT *Body Uneasiness Test; *EDE-Q* Eating Disorder Examination Questionnaire; *GSI* Global Severity Index; *IDEA* Identity and Eating Disorders; *SCL-90-R* Symptom Checklist 90-Revised; *TS* total score

Table 1 shows the longitudinal trend of the psychometric variables. A statistically significant reduction in the total scores of all psychometric scales was observed at follow-up after the first year of treatment (all *p* < 0.05) (Table 1), in all the observed sample. This reduction was also maintained at the second follow-up, carried out 1 year after the first, as the Bonferroni-corrected post hoc pairwise comparisons between T0 and T1 and between T0 and T2 were all statistically significant (all *p* < 0.05) (Table 1), while no comparison between T1 and T2 was significant (all *p* > 0.05). The longitudinal course of these variables is shown in Fig. 1. The trend was similar for almost all subscales of the questionnaires, except for BUT avoidance and IDEA GEO for participants with AN, and IDEA S for those with BN, which showed a significant reduction compared to the baseline only at T2 (*p* < 0.05) (Table 1).

A similar model was carried out for BMI. Post-hoc tests revealed significant differences between T0 and T1 and between T0 and T2 only for patients with AN (*p* < 0.05), while no significant differences were found between T1 and T2 (*p* > 0.05) (Table 1). BMI did not vary significantly in the BN group (all *p* > 0.05).

Considering the whole sample, 24 participants (17.0%) were found to have experienced a diagnostic crossover at the last follow-up: 3 to AN, 7 to BN and 14 to Binge-eating disorder. Of those who at T2 did not show diagnostic crossover, 63 (44.7%) obtained remission (34 partial remissions and 29 full remissions) according to the DSM-5 criteria, of which 22 originally had AN and 41 BN. Multivariate binomial logistic regression showed that baseline IDEA total score and BN diagnosis were associated with a higher probability of diagnostic crossover at T2 (χ^2^(2) = 8.76, *p* = 0.013; IDEA total score OR: 1.80 [1.01–3.20], *p* = 0.045; BN diagnosis OR: 2.84 [1.06–7.62], *p* = 0.038).

#### Longitudinal mediation model

A two-instance repeated-measures mediation analysis was performed to estimate the direct and indirect effects of the within-subjects independent variable, Time, on the difference in ED psychopathology and body uneasiness through the variation of the mediator, the embodiment disorder.

**Fig. 2:**
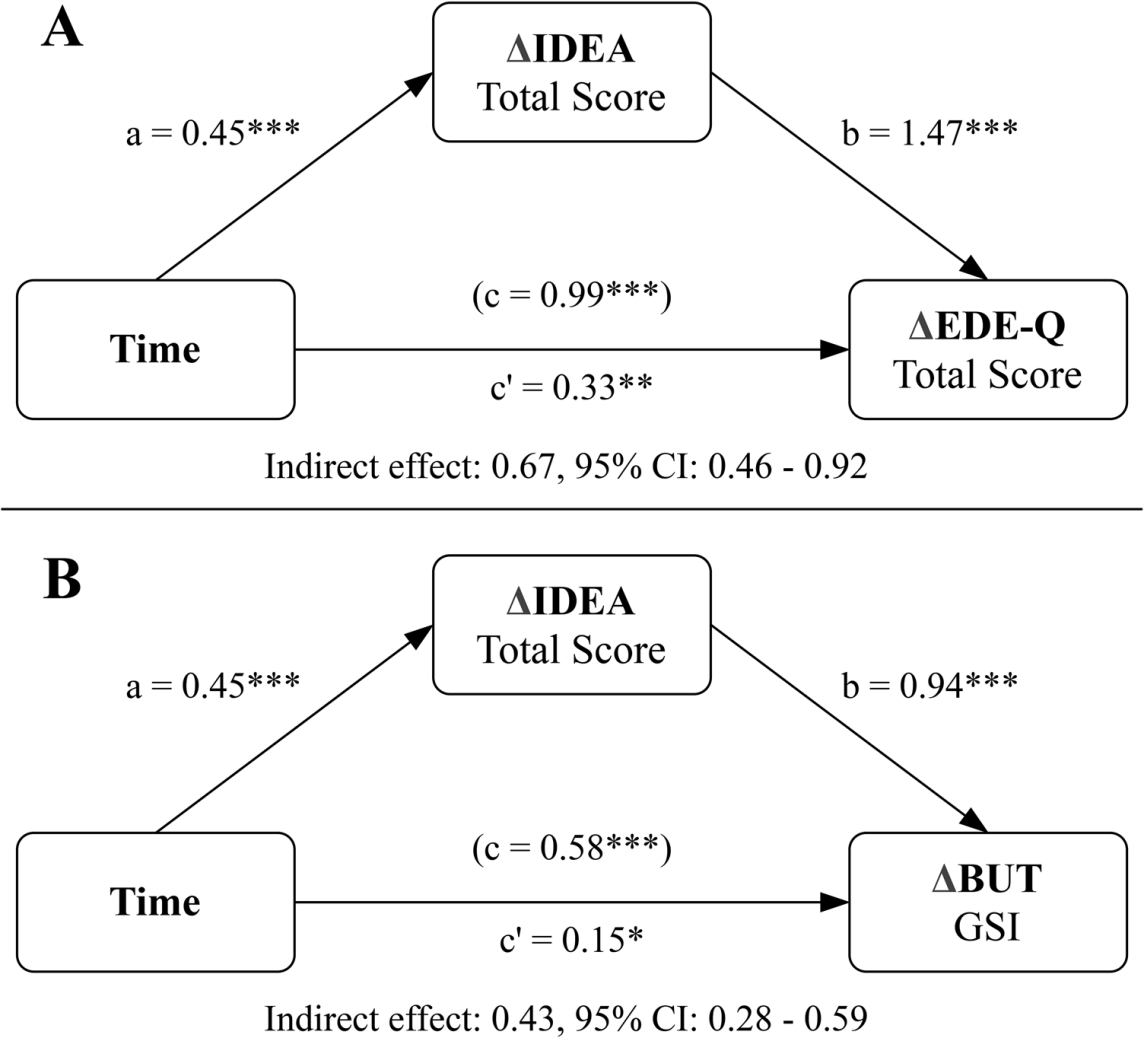
Longitudinal mediation analyses for EDE-Q (panel **a**) and BUT (panel **b**). T2–T0 variations are indicated with Δ. Both the total (*c*) and direct effect (*c*′) of the independent variable (time) on the dependent variable are reported. *BUT* Body Uneasiness Test; *CI* confidence interval; *EDE-Q* Eating Disorder Examination Questionnaire; *GSI* Global Severity Index; *IDEA *Identity and Eating Disorders. *p < 0.05; **p < 0.01; ***p < 0.001

Time had a significant effect on the variation of EDE-Q TS (Fig. 2a, path *c*), BUT GSI (Fig. 2b, path *c*) and IDEA TS (Fig. 2, paths *a*). The variation of the mediator significantly predicted both outcomes variables, even when adjusting for time (Fig. 2, paths *b*). Bootstrapping analysis showed significant indirect effects of time through IDEA TS on both ED-specific psychopathology (Fig. 2a) and body uneasiness (Fig. 2b). However, Time maintained its statistical significance even with the mediator as a covariate (Fig. 2, paths *c’*), indicating a partial mediation with significant direct effects.

## Discussion

To the best of our knowledge, this is the first study in the field of clinical phenomenology attempting to elucidate the role of the embodiment disorder, as measured by the questionnaire IDEA [4], in the treatment of EDs.

Longitudinal data showed that after 1 year of multidisciplinary treatment, which included CBT-E, both patients with AN and BN underwent a significant improvement of general and ED-specific psychopathology and body uneasiness, which remained stable at the second follow-up performed 1 year after the first one. Interestingly, for the first time this study demonstrated that treatment also determined a stable reduction of the embodiment disorder. Overall, these data confirm the efficacy of CBT-E in the treatment of EDs. However, the remission rate was far from satisfactory, considering that less than a half of all patients reported a partial or full remission, substantially in line with what previously observed by many other studies in this field [21, 22]. Moreover, about one out of five patients experienced diagnostic crossover, confirming the high prevalence of diagnostic instability in patients with EDs [28, 29]. Regarding the role of the embodiment disorder as a possible outcome predictor, data showed that it was positively associated with diagnostic crossover. This result is in line with previous findings concerning the association between diagnostic instability and various indicators of disease severity, such as adverse early life experiences [30], concurrent psychiatric diagnosis [31] or low self-directedness [29]. Moreover, the relationship between impaired lived corporeality and the transition between different diagnostic categories confirms the transdiagnostic nature of this psychopathological dimension and its crucial role in the spectrum of EDs [4].

The longitudinal mediation model showed that the amelioration of the embodiment disorder mediated both the improvement of body uneasiness and the erosion of the classic symptoms of EDs represented by the overinvestment on body shape/weight. For the first time, these results suggest that the embodiment disorder is not just a *core* psychopathological feature in EDs, as previously observed [4, 5, 7, 32, 33], but that it is also involved in maintaining these disorders, with a potentially crucial role as a target of intervention. As expected, the direct effect of time was also significant, suggesting that the variation of the embodiment disorder was not the only mechanism involved in the amelioration of classic ED symptoms. Nevertheless, it is to be noted that the indirect effect comprehending the role of the variation of the embodiment disorder was much greater than the direct effect of time. These findings are in line with the studies that underline the importance of addressing the abnormal bodily experiences of patients with EDs, particularly interoceptive deficits, in treating these disorders [34]. Indeed, according to the optical-coenaesthesic disproportion hypothesis, interoceptive deficits are crucial aspects in the phenomenology of the embodiment disorder in EDs [6]. In particular, as a consequence of the impaired coenaesthesia, patients with EDs feel extraneous from their own body, are unable to trust it, have an altered perception of time which leads them to think that in the twinkling of an eye their own body may become as big as an air balloon [4, 35]. Consequently, they are locked in an allocentric representation of their body [36] and they over-rely on the gaze of the others and their pathological eating behaviours as the only ways through which they can define their self [7]. It could be hypothesised that the reason why the amelioration of the embodiment disorder determines a reduction of the overvaluation of body shape/weight and body uneasiness might be the fact that the renewed confidence in the stability of their own body in space and time and the restoration of their identity might help patients to start looking again beyond their corporeality, widening their horizon of values [37].

CBT-E is first and foremost aimed at interrupting abnormal behaviours such as starvation, binge-eating and body checking through the disputing of the distorted beliefs related to body shape and weight [2]. Although it does not include specific embodiment-focused modules, it could be hypothesised that it might help patients recover a healthier lived corporeality because of the so-called non-specific therapeutic factors, such as therapeutic alliance, empathy and related constructs, and patients’ expectations [38, 39]. On the other side, it is possible that the efficacious effect of CBT-E in reducing pathological eating behaviours might improve the impaired leaved corporeality, even though CBT-E is not directly targeted to this dimension. Indeed, weighting oneself several times a day, compulsively checking one’s body parts, or believing that bodily dimensions might abruptly change after ingesting a single candy are all features that might be involved in maintaining the impaired capacity of feeling one’s bodily sensations (bottom-up effect).

However, the improvement of CBT-E with phenomenological concepts, namely with the optical-coenaesthesic disproportion hypothesis, may contribute to overcoming some of its limitations, making it more effective. Indeed, the integration with phenomenologic modules focused on the exploration of domains of selfhood and identity (top-down strategies), that go beyond the conceptualisation of ED symptoms as consequences of the overvaluation of body shape and weight, may contribute to help patients to reconstruct their personal identity [40], overcoming the identification of their Self with the status of “anorexic person” [4, 6]. Moreover, the implementation of modules specifically aimed at addressing the interoceptive deficits of these patients [34] might be a precious resource in the complicated process of recovering a healthier contact with one’s bodily sensations and thus with emotions [41], consequently breaking the pathological interconnection between impaired coenaesthesia, imprisonment in an allocentric perspective on one’s own body [6, 36] and overreliance on the gaze of the others as the only way through which defining one’s own identity. Furthermore, considering the crucial role of insecure attachment style and more in general of interpersonal problems in the development of the embodiment disorder and abnormal bodily experiences in patients with EDs [42, 43], with *the other* becoming a defining glance for an undefined body rather than the object of a constructive interaction [4], it would be of great clinical interest to consider the implementation of modules focused on evolutionary and relational aspects aimed at restoring a healthier dialogue between selfhood and alterity [44].

As an overall remark and as a suggestion for further research, the authors of the present study believe that cognitive, phenomenological and psychodynamic approaches should not be considered separate and mutually exclusive. The cognitive-behavioural intervention is certainly the one with more empirical evidence. However, clinical observations seem to suggest that the contamination and integration of the cognitive model with the more comprehensive phenomenological view could be useful for the long-term outcome of the recovery process. In line with this perspective, a comprehensive assessment of patients with EDs should investigate the way of perceiving one’s own body and lived corporeality, the role of the illness and the body in inter-subjective interactions, as well as the personal identity definition, the spatial perception and the way of experiencing time associated with several ED features (such as binge eating or weight control). All this would make it possible to obtain an integrated psychotherapeutic model targeting not just the cognitive-ideational dimensions of ED psychopathology, but also the experiential, perceptual, emotional, identity-related and relational aspects that together constitute the roots and the matter of these diseases.

In conclusion, the present study underlined the crucial role of the improvement of the embodiment disorder as a mediator of the efficacy of multidisciplinary treatment including CBT-E for patients with AN and BN. This finding has several implications. First of all, it corroborates the hypothesis that a disturbed lived corporeality may represent a deeper layer in the conceptualization of EDs, where the classic ED symptoms draw their roots. Moreover, for the first time this study proposes an improvement of cognitive-behavioural techniques by phenomenological models of AN and BN for the treatment of these disorders, suggesting that it would be useful to integrate CBT-E with specific phenomenology-based, embodiment-focused modules to increase its effectiveness in the treatment of EDs.

### Strength and limits

The major strength of the present study is that for the first time it shows the role of the embodiment disorder as a mediator of the improvement of classic ED symptoms in patients treated with CBT-E through a longitudinal design with a 2-year follow-up. However, it also has some limitations. First, the sample size was limited. Second, all psychopathological domains were investigated only through self-administered questionnaires. Third, it should be noted that the follow-up period partially overlapped with the COVID-19 outbreak in Italy. However, this could hardly have influenced the results given the limited number of follow-ups carried out after the start of the pandemic (*n* = 5). Furthermore, the COVID-19 outbreak was not associated with significant worsening of ED-specific psychopathology in a recent study on ED patients [45]. Finally, some patients were taking drug treatment for concurrent psychiatric symptomatology during the study. However, given the limited number of this subgroup of participants, this is unlikely to have altered the results significantly. Furthermore, there is currently no evidence of the effect of drug therapies on ED psychopathology in patients with AN [46], and there is only limited data regarding fluoxetine in BN [47].

## What is already known on this subject?

Available data show that the outcome of patients with AN or BN treated with CBT-E is far from being satisfactory, with a long- term remission rate which is less than 50%. This might be the consequence of the paucity of evidence regarding the mediators of the efficacy of treatment interventions on EDs psychopathology, both in terms of body uneasiness and of overvaluation of body shape/weight. Following numerous studies suggesting that the embodiment disorder is a core feature of eating disorders, this study focused on investigating its role as a possible mediator of treatment outcome.

## What your study adds?

The present study highlighted the crucial role of the embodiment disorder as a mediator of the efficacy of a multidisciplinary treatment including CBT-E for patients with AN and BN. This finding corroborates the hypothesis that a disturbed lived corporeality may represent a deeper layer in the conceptualization of EDs. Moreover, for the first time this study proposes an improvement of cognitive-behavioural techniques by phenomenological models of AN and BN for the treatment of these disorders, suggesting that it would be useful to integrate CBT-E with specific phenomenology-based, embodiment-focused modules to increase its effectiveness in the treatment of EDs.

## Availability of data and material

Research data are not shared.

## Data Availability

Not applicable.
